# Hepatitis E Virus Genotype 3 in Shellfish, United Kingdom

**DOI:** 10.3201/eid1812.120924

**Published:** 2012-12

**Authors:** Claire Crossan, Paul J. Baker, John Craft, Yasu Takeuchi, Harry R. Dalton, Linda Scobie

**Affiliations:** Author affiliations: Glasgow Caledonian University, Glasgow, Scotland, UK (C. Crossan, P.J. Baker, J. Craft, L. Scobie);; University College London, London, UK (Y. Takeuchi);; European Centre for Environment and Human Health, University of Exeter Medical School, Truro, UK (H.R. Dalton)

**Keywords:** hepatitis E, hepatitis E virus, shellfish, zoonoses, viruses, bivalve mollusks, bivalve molluscs, United Kingdom

**To the Editor**: Bivalve mollusks (shellfish), such as mussels and oysters, are filter feeders; they concentrate microorganisms of human and animal origin (up to 100×) from the surrounding environment. Several recent reports have linked the incidence of human infection with hepatitis E virus (HEV) to consumption of undercooked pork, game products, and shellfish (*1*,*2*). Infectious HEV has been found in swine manure and wastewater (*3*); therefore, application of manure to land and subsequent runoff could contaminate coastal water, leading to contamination of shellfish and, subsequently, possible human infection. Because they are filter feeders, bivalve mollusks are biologically relevant sentinels and can indicate potential pathogens that are contaminating the environment. It is essential to ensure that this sustainable resource of coastal areas, where mussels and oysters are farmed or collected wild, is not subjected to environmental contamination that could lead to public health risks.

Risk management for bivalve mollusks, aimed at control of fecal pollution, relies heavily on the use of *Escherichia coli* as an indicator of fecal (sewage) contamination and is enacted under European food regulations (Regulation 854/2004, www.cefas.co.uk/media/455777/extract_reg_no_854_2004.pdf). However, although these regulations probably reduce the number of infections, especially bacterial infections, they are not viewed as adequately controlling the risk for viral infections. Specific risks are posed by the robustness of viruses in the environment and the different behavior of viruses within bivalve mollusks compared with behavior within bacterial fecal indicators. 

HEV is deemed to be inactivated during processing procedures used to prepare mussels for consumption; however, HEV is only 50% inactivated at 56°C and 96% at 60°C for 1 hour, it is stable when exposed to trifluorotrichloroethane, and it is resistant to inactivation by acidic and alkaline conditions (*4*). Most shellfish are usually eaten raw, but viable virus can also pose a risk to public health in shellfish that are lightly steamed or preserved by smoking and/or in acetic acid. Indeed, a recent study by the Food Standards Agency, in which >800 oyster samples from 39 growing beds in the United Kingdom were collected and screened during 2009–2011, found norovirus at low levels in at least 76% of oysters (*5*). Other studies identified hepatitis A virus and norovirus in shellfish production areas and in ready-to-eat products in the United Kingdom (*1*,*6*). In fact, depuration experiments demonstrated no decrease in titers against hepatitis A virus over a 23-hour cleansing period (*7*). In addition, acute HEV infection attributed to consumption of shellfish was diagnosed for 33 passengers who recently returned from a cruise (*2*). However, data have been restricted to questionnaires implicating consumption of shellfish as a source of transmission; no follow-up analyses of the contaminated foodstuff have been conducted. Thus, possible transmission routes for HEV remain poorly studied in the United Kingdom (*2*).

To determine whether HEV is present in mussels collected locally for human consumption, we examined 48 mussels from 5 tidal locations in Scotland. We collected closed mussels from the west coast of Scotland (11 at Lunderston Bay and 28 at Ardrossan) and the east coast of Scotland (9 at Stannergate, Dundee; Ferryden, Montrose; and the Ythan Estuary at Newburgh). 

The site at Ardrossan was near a slaughterhouse and a meat preparation purification plant that processes pigs. The plant was considered a potential source of contamination, and mussels were collected in a 10-m^2^ area around an outfall (drain/sewage pipe) directly in line with the processing plant.

A total of 36 (92%) of the 39 mussels from the west coast were positive by PCR for HEV, and 5 (55%) of the 9 from the east coast were positive. The mean value of HEV RNA detected in the samples was 4.25 log_10_ IU/mL (range 3.73–5.2 log_10_ IU/mL), and the assay was validated by using the current candidate HEV World Health Organization standard (http://whqlibdoc.who.int/hq/2011/WHO_BS_2011.2175_eng.pdf). Phylogenetic analysis showed that most bivalve mollusk sequences clustered with HEV genotype 3 from humans and swine (Figure; Technical Appendix). Also, HEV sequences isolated specifically from a UK human source corresponded with sequences isolated from the bivalve mollusks. The presence of a swine-like HEV genotype 3 in freshwater bivalve mollusks has also been reported in Japan and South Korea (*1**,**9*). 

**Figure:**
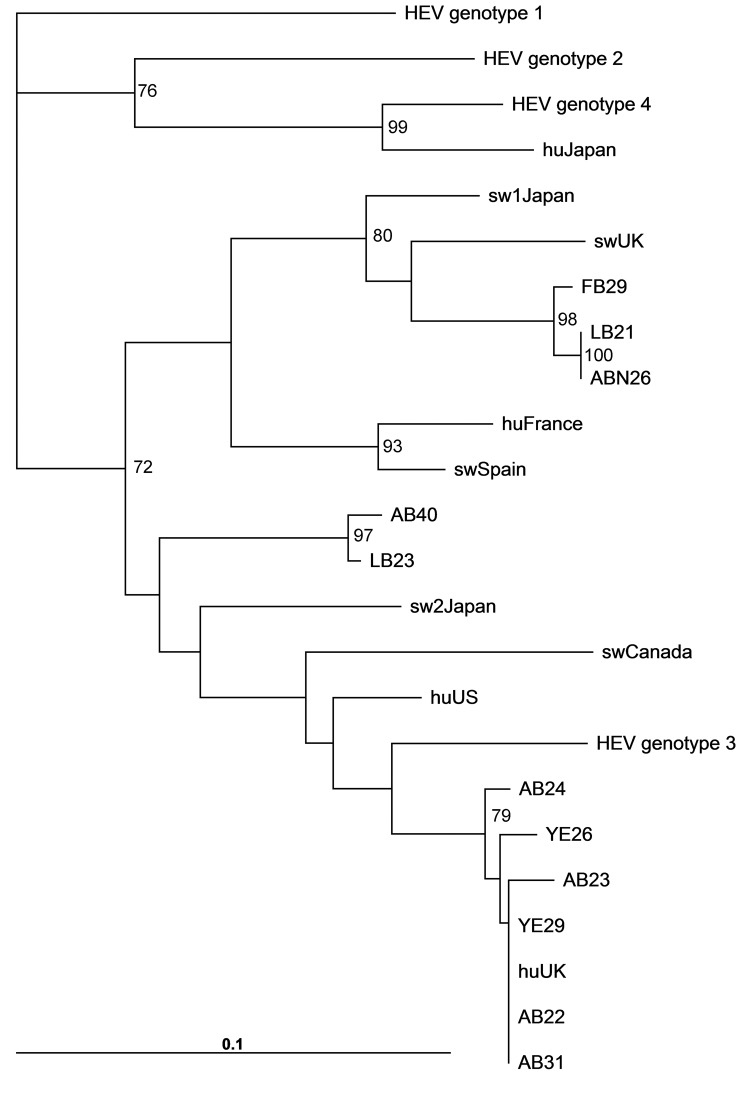
Phylogenetic analysis of HEV open reading frame 2 sequences isolated from *Mytilus* spp. RNA was isolated from 50–100 mg of digestive gland or gill. Tissue was homogenized in 300 μL phosphate-buffered saline, and viral RNA was isolated by using a viral RNA kit (QIAGEN, Crawley, UK), and PCR was conducted by amplifying nucleotides 6332–6476 as described (*8*). The nucleotide sequences were aligned and bootstrapped, and phylogenetic neighbor-joining trees were constructed by using the ClustalW software (www.ebi.ac.uk/Tools/msa/clustalw2/). Phylogenetic trees were visualized by using FigTree (http://tree.bio.ed.ac.uk/software/figtree/). Bootstrap values >70% are indicated. Scale bar indicates nucleotide substitutions per site. Sample site codes: AB, Ardrossan Beach; LB, Lunderston Bay; ABN, Aberdeen; FB, Ferrybridge; YE, Ythan Estuary. Sequences: Sw, swine; hu, human (followed by country of origin). GenBank accession numbers for reference sequences: HEV genotype 1, B73218; HEV genotype 2, M74506; HEV genotype 3, CO31008; HEV genotype 4, C272108; huUK (KernowC1), HQ389543; HuUS, JN837481; swUK AF503512; huFrance, JN906974; swCanada, AY115488; swSpain, JQ522948; sw2Japan AB248521, huJapan AB161719.

Worldwide, an estimated 40,000 persons die and another 40,000 experience long-term disability as a result of consuming raw or undercooked shellfish (*10*). This study, demonstrating the presence of HEV in mussels collected locally in Scotland for human consumption, raises concern as to whether these shellfish are a potential source of infection, as reported (*2*). The association between environmental contamination with HEV and possible transmission by eating shellfish warrants investigation.

Technical Appendix ClustalW alignment of sequences used to generate the phylogenetic tree in the Figure.
