# Supplementary material for: Hepatitis E Virus Genotype 3 in Shellfish, United Kingdom
Source: Emerg Infect Dis. 2012 Dec;18(12):2085–7. doi: 10.3201/eid1812.120924 (PMC3557861; doi:10.3201/eid1812.120924)
Supplement: Technical Appendix — ClustalW alignment of sequences used to generate the phylogenetic tree in the Figure. [file 12-0924-Techapp-s1.pdf]

Article DOI: http://dx.doi.org/10.3201/eid1812.120924

# Hepatitis E Virus Genotype 3 in Shellfish, United Kingdom

## Technical Appendix

```
LB21           CGACTGTTAAATTATACACCTCCGTTGAGAATGCACAGCAGGATAAGGGCATTGCCATAC 60
ABN26          CGACTGTTAAATTATACACCTCCGTTGAGAATGCACAGCAGGATAAGGGCATTGCCATAC 60
FB29           CGACTGTTAAATTATACACCTCCGTTGAGAATGCACAGCAGGATAAGGGCATTGCCATAC 60
swUK           CGGCTGTTAAATTATATACTTCCGTCGAGAATGCACAGCAGGATAAGGGCATTGCCATAC 60
huFrance       CAACTGTTAAGTTATACACCTCTGTCGAGAATGCACAGCAGGATAAGGGTATTGCTATAC 60
AB40           CGACTGTCAAATTATACACATCTGTCGAGAATGCACAGCAGGACAAGGGCATTGCCATAC 60
LB23           CGACTGTCAAATTATACACATCTGTCGAGAATGCGCAGCAGGACAAGGGCATTGCCATAC 60
AB7            CGACAGTAAAGTTATATACATCTGTAGAGAATGCGCAGCAAGACAAGGGCATTACCATCC 60
YE26           CGACAGTAAAGTTATATACATCTGTAGAGAATGCGCAGCAAGACAAGGGCATTACCATCC 60
YE29           CGACAGTAAAGTTATACACATCTGTAGAGAATGCGCAGCAAGACAAGGGCATTACCATCC 60
huUK           CGACAGTAAAGTTATACACATCTGTAGAGAATGCGCAGCAAGACAAGGGCATTACCATCC 60
AB31           CGACAGTAAAGTTATACACATCTGTAGAGAATGCGCAGCAAGACAAGGGCATTACCATCC 60
AB22           CGACAGTAAAGTTATACACATCTGTAGAGAATGCGCAGCAAGACAAGGGCATTACCATCC 60
AB23           CGACAGTAAAGTTATACACATCCGTAGAGAATGCGCAGCAAGACAAGGGCATTACCATCC 60
AB24           CGACAGTAAAGTTATACACATCTGTAGAGAATGCGCAGCAAGACAAGGGCATTACCATTC 60
HEVgenotype3   CAACAGTAAAGTTATACACATCTGTTGAGAATGCGCAGCAAGACAAGGGCATCACCATTC 60
huUS           CAACAGTAAAGTTATATACATCTGTTGAGAATGCGCAGCAAGACAAGGGCATTACCATCC 60
HEVgenotype1   CGACTGTTAAGTTGTATACATCTGTAGAGAATGCTCAGCAGGATAAGGGTATTGCAATCC 60
HEVgenotype2   CAACCGTGAAGCTCTATACATCAGTGGAGAATGCTCAGCAGGATAAGGGTGTTGCTATCC 60
HEVgenotype4   -GACAGTGAAACTTTACACTTCAGTCGAGAACGCTCAGCAGGACAAGGGTGTAGCTATTC 59
                  * ** **  * ** ** ** ** ***** ** ***** ** *****  *  * ** *

LB21            CTCATGATATAGACTTAGGGGACTCTCGCGTGGTTATC 98
ABN26           CTCATGATATAGACTTAGGGGACTCTCGCGTGGTTATC 98
FB29            CTCACGATATAGACTTAGGGGACTCTCGCGTGGTTATC 98
swUK            CTCACGATATAGACCTAGGGGATTCCCGCGTGGTTATC 98
huFrance        CACACGATATAGACCTAGGGGATTCCCGTGTGGTTGTA 98
AB40            CACATGATATAGATCTGGGAGATTCTCGTGTGGTTATT 98
LB23            CACATGATATAGATCTGGGAGATTCTCGTGTGGTTATT 98
AB7             CACACGATATAGATTTGGGTGATTCCCGTGTGGTTATT 98
YE26            CACACGATATAGATTTGGGTGATTCCCGTGTGGTTATT 98
YE29            CACACGATATAGATTTGGGTGATTCCCGTGTGGTTATT 98
huUK            CACACGATATAGATTTGGGTGATTCCCGTGTGGTTATT 98
AB31            CACACGATATAGATTTGGGTGATTCCCGTGTGGTTATT 98
AB22            CACACGATATAGATTTGGGTGATTCCCGTGTGGTTATT 98
AB23            CACACGATATAGATTTGGGTGATTCCCGTGTGGTTATT 98
AB24            CACACGATATAGATTTGGGTGATTCCCGTGTGGTTATT 98
HEVgenotype3    CACACGACATAGATTTAGGTGACTCCCGTGTGGTTATC 98
huUS            CACATGATATAGATCTGGGTGACTCCCGTGTGGTTATC 98
HEVgenotype1    CGCATGACATTGACCTCGGAGAATCTCGTGTGGTTATT 98
HEVgenotype2    CCCACGATATCGATCTTGGTGATTCGCGTGTGGTCATT 98
HEVgenotype4    CACATGATATTGACCTTGGTGAGTCCCGTGTGGTTATT 97
                * ** ** ** **  * ** ** ** ** *****  *
```

Technical Appendix Figure. ClustalW alignment of sequences used to generate the phylogenetic tree in the Figure (see article text). Sequences were generated from RNA isolated from 50–100 mg of digestive gland or gill. Tissue was homogenized in 300 μL phosphate-buffered saline, and viral RNA was isolated by using a viral RNA kit (QIAGEN, Crawley, UK), and PCR was conducted by amplifying nucleotides

6332–6476 as described (*1*). The nucleotide sequences were aligned and bootstrapped, and phylogenetic neighbor-joining trees were constructed by using the ClustalW software (www.ebi.ac.uk/Tools/msa/clustalw2).

**Reference**

1. Erker JC, Desai SM, Mushahwar IK. Rapid detection of hepatitis E virus RNA by reverse transcription–polymerase chain reaction using universal oligonucleotide primers. J Virol Methods. 1999;81:109. PubMed http://dx.doi.org/10.1016/S0166-0934(99)00052-X
